# Unveiling the Mask: Gender Dysphoria and the Path to Holistic Healing

**DOI:** 10.7759/cureus.76738

**Published:** 2025-01-01

**Authors:** Isabel C Tavares, Pedro A Figueiredo

**Affiliations:** 1 Family Medicine, Unidade Local de Saúde Lisboa Ocidental - Unidade de Saúde Familiar Conde de Oeiras, Oeiras, PRT; 2 Family Medicine, Unidade Local de Saúde Santa Maria - Unidade de Saúde Familiar Rodrigues Miguéis, Lisboa, PRT

**Keywords:** depressive disorder, doctor-patient relationship, gender dysphoria, holistic healthcare, mental health, metabolic syndrome, obesity

## Abstract

Gender dysphoria and gender incongruence occur when an individual’s gender identity differs from their biological sex, often resulting in significant distress. This report examines a 34-year-old male patient with severe obesity, depression, metabolic syndrome, and cardiovascular disease, who visited his family doctor in June 2021 following a recent diabetes diagnosis. Notably, the patient was newly recognized as experiencing gender dysphoria, which is believed to have been present for many years. This previously unrecognized dysphoria may have contributed to various comorbidities throughout his life, including social isolation, depression, obesity, and cardiovascular disease. The case highlights the importance of a holistic healthcare approach, emphasizing the integration of social context, mental health, and gender identity with physical health considerations. This comprehensive perspective enabled the physician to identify a potential underlying factor contributing to the patient’s medical challenges. The case also illustrates the interconnectedness of mental and physical health and underscores the value of a strong doctor-patient relationship, which created a safe space for the patient to disclose his long-held gender dysphoria for the first time.

## Introduction

The current criteria for diagnosing gender incongruence involve persistent incongruence between an individual’s gender identity and their sex assigned at birth, which is typically determined based on external sexual anatomy. This diagnosis is essential for considering gender-affirming hormones and surgical therapies. Additionally, individuals may be diagnosed with gender dysphoria, defined as the distress stemming from this incongruence rather than the incongruence itself. Diagnostic criteria are primarily guided by the Diagnostic and Statistical Manual of Mental Disorders, Fifth Edition (DSM-5), which emphasizes persistent discomfort with the mismatch between gender identity and external anatomy, as well as significant interference in areas such as social or academic functioning [1,2].

Data from the United States suggest that approximately 1.4% of the youth population and 0.5% of the adult population identify as transgender [3]. However, precise global figures remain elusive, complicating healthcare planning. Transgender individuals represent a diverse population, encompassing those who live with gender incongruence without transitioning, those who transition socially without seeking medical intervention, and many who face barriers to accessing care due to stigma or reliance on nonmedical resources. Consequently, research often focuses on individuals accessing gender-affirming care at specialized clinics, thereby underestimating the broader transgender population and excluding those who might benefit from nonclinical resources like information and counseling. A recent study extrapolated the 0.5% prevalence rate to the global population aged 15 years and older, estimating approximately 25 million transgender individuals worldwide [4].

In Portugal, accurate data on the prevalence of transgender individuals are limited, with a lack of comprehensive demographic studies targeting this population. A 2023 review underscored the scarcity of research in this area, highlighting the need for more systematic data collection to better understand and support the transgender community [5].

Individuals with gender dysphoria are at a significantly higher risk of developing mental health conditions such as anxiety, depression, and eating disorders [6]. The psychological distress associated with gender dysphoria, especially when compounded by depression, can trigger both psychological and biological stress responses. These responses can lead to physical health comorbidities, including metabolic syndrome and cardiovascular disease. This underscores the complex, bidirectional relationship between mental and physical health. Addressing this interplay is essential for optimizing healthcare outcomes for transgender individuals [7,8].

## Case presentation

We present the case of a 34-year-old Caucasian male with a medical history of severe obesity (BMI: 58.1 kg/m²), deep vein thrombosis, pulmonary thromboembolism, and major depressive disorder. His parents divorced during his puberty, resulting in a strained relationship and limited social support. The patient had never held a job and presented to his family doctor (FD) in June 2021 following a recent diagnosis of diabetes. At this appointment, he reported long-term symptoms, including social isolation, clinophilia, hyperphagia, hypersomnia, and anhedonia.

The primary care physician proposed psychological support and repeatedly suggested antidepressant medication; however, the patient declined pharmacological treatment. Following effective diabetes management, the patient underwent bariatric surgery in July 2022. This intervention led to significant weight loss (BMI reduced to 36 kg/m² by April 2023), relief from symptoms such as dyspnea and fatigue, and complete reversal of diabetes (HbA1c improved from 7.9% at diagnosis to 5.9%). These changes marked a noticeable improvement in his self-esteem and depressive symptoms, representing a turning point in his life.

In May 2023, the patient scheduled another appointment with his FD, during which he disclosed longstanding symptoms related to gender incongruence. He traced these feelings back to early childhood, describing a persistent sense of being different and discomfort with his male gender. He reported avoiding behaviors traditionally associated with masculinity and noted that he had never disclosed these experiences before. He acknowledged that the recent physical changes following bariatric surgery had triggered a desire to embrace his true identity and start living authentically.

Based on the patient’s history and symptoms, a diagnosis of gender dysphoria was made (Table 1), consistent with DSM-5 criteria: (1) a marked incongruence between experienced/expressed gender and assigned gender at birth, persisting for more than six months; (2) a strong desire to be treated as a gender other than assigned at birth; and (3) significant distress or impairment in functioning. His longstanding social isolation, depressive symptoms, and avoidance behaviors were identified as manifestations of this distress. The patient was referred to the sexology department at the reference hospital for further evaluation and management.

**Table 1:** Symptoms described by the patient and corresponding diagnosis (DSM-5) All symptoms that result in clinically significant impairment in key areas of functioning, including social, academic, and occupational domains. DSM-5, Diagnostic and Statistical Manual of Mental Disorders, Fifth Edition

Diagnosis	Symptoms
Major depressive disorder	Depressed mood
	Anhedonia
	Weight change/hyperphagia
	Hypersomnia
	Social isolation
	Fatigue/clinophilia
Gender dysphoria	Incongruence between gender identity and gender assigned at birth
	Strong conviction that has feelings and behaviors more typical of the other gender
	Strong desire to be the other gender (self-identification as another gender)
	Strong desire to be rid of secondary sex characteristics

In January 2024, eight months after the referral, the patient was evaluated by a psychiatrist, where nonpharmacological measures, such as promoting autonomy and self-determination, were discussed. The patient was also guided and educated about the process of disclosure. During the following appointment, the patient expressed a desire to be treated as female. As such, the pronoun “she” will be used for the remainder of the paper.

As of April 2024, there has been a clear improvement in self-care and mood, with reduced stress and a noticeable decrease in depressive symptoms. She feels enthusiastic and hopeful about the process and her future. While she has expressed a desire to begin hormonal therapy, this has not yet been initiated. She continues to follow up with her FD, bariatric surgeon, and sexologist, remaining determined to improve her overall health and habits.

## Discussion

This case underscores the intricate interplay between mental and physical health, particularly in the context of sexual and gender identity. Observational studies indicate that sexual minority youth experience higher rates of mental health issues, including anxiety, depression, suicidality, and substance use, compared to their heterosexual peers [9,10]. Prolonged exposure to negative experiences during childhood and adolescence has been proposed as a psychological stressor that may activate the body’s stress response, leading to adverse physical and mental health outcomes later in life.

Emerging evidence suggests that chronic stress can elevate pro-inflammatory cytokines and CRP levels, contributing to systemic inflammation. This physiological state has been linked to hyperphagia, sleep disturbances, hypertension, and visceral fat accumulation, all of which increase the risk of cardiovascular disease, obesity, and metabolic syndrome over time [8]. In this case, chronic psychological stress - partly due to unrecognized gender dysphoria and its associated depressive disorder - likely contributed to the patient’s sedentary lifestyle and weight gain. These factors, combined with the systemic effects of chronic stress, played a significant role in the development of severe obesity and its related complications. This highlights the multifactorial nature of the patient’s condition and the bidirectional relationship between untreated gender dysphoria, mental health challenges, and physical comorbidities.

A lack of competence and training among healthcare providers, coupled with persistent stigma within healthcare settings, is thought to contribute to the disparities faced by sexual minorities compared to the general population. Sexual minorities often delay seeking medical care and frequently lack a consistent healthcare provider. While numerous factors contribute to health disparities in this population, empirical evidence suggests that stigma, discrimination, and mistreatment in healthcare settings frequently deter sexual minorities from accessing the care they need [11]. Healthcare providers play a pivotal role in fostering openness and support for diversity, offering valuable information about community resources, and creating safe spaces for families to address concerns and overcome fears. FDs, in particular, can facilitate early identification of gender-related issues during childhood and adolescence, enabling timely supportive interventions for both individuals and families.

Personal and familial acceptance of sexual identity is a crucial protective factor for sexual minorities, as it has been shown to mitigate some adverse outcomes often seen in sexual minority youth [12]. Numerous observational studies demonstrate that caregiver support and acceptance correlate with positive mental health outcomes, whereas caregiver rejection and inadequate support are linked to negative outcomes [13,14]. Protective factors such as acceptance, high self-esteem, psychological well-being, strong self-identity, ethnic identification, and robust connections to family, school, and community can help reduce stigmatization and enhance resilience [15-18]. In this case, many of these protective factors were notably absent, contributing to the complexity of the patient’s condition.

The diagnosis of a chronic illness, such as diabetes mellitus, created an opportunity for regular follow-up with the FD and facilitated discussions about the patient’s overall health and well-being. These consistent appointments, along with the physical changes following bariatric surgery, allowed for a gradual exploration of underlying issues, culminating in the eventual disclosure of gender dysphoria. However, it is important to acknowledge that socioeconomic and sociocultural factors, which may influence the diagnosis and management of gender dysphoria, were not formally assessed in this case. These limitations highlight the need for further research to better understand the multifaceted contributors to health outcomes in individuals with gender dysphoria.

This case also highlights the uniqueness of its findings, as no similar cases linking gender dysphoria with multiple chronic pathologies have been reported in Portugal. It emphasizes the importance of healthcare providers recognizing the connections between mental health, gender identity, and physical health and fostering supportive environments to address these intertwined challenges. A holistic approach and sustained care were pivotal in facilitating the identification and management of gender dysphoria in this patient.

## Conclusions

Gender dysphoria shares parallels with other forms of chronic stress, often contributing to both psychological and physical health challenges. Although the mechanisms linking chronic stress to health outcomes are complex and multifaceted, this case highlights the intricate interplay between mental health, gender identity, and physical well-being.

This report underscores the critical need to raise awareness among clinicians about the unique healthcare needs and barriers experienced by sexual minorities. In this case, regular follow-up visits and the physical health improvements achieved through bariatric surgery provided a supportive context for the patient to disclose long-standing feelings of gender dysphoria. General practitioners play a vital role in fostering safe and supportive environments that enable the exploration and management of underlying issues, such as gender dysphoria, thereby facilitating improved health outcomes.
